# Assessment of the young adult hip joint using plain radiographs

**DOI:** 10.1007/s12306-020-00650-2

**Published:** 2020-03-03

**Authors:** R. Popat, S. Lee, D. A. George, D. Amiras, K. M. Sarraf

**Affiliations:** 1 West Hertfordshire Hospitals NHS Trust, Watford General Hospital, Vicarage Road, Watford, Hertfordshire, WD18 0HB UK; 2 Imperial College Healthcare NHS Trust, St Mary’s Hospital, Praed Street, London, W2 1NY UK; 3 Department of Trauma and Orthopaedic Surgery, West Hertfordshire Hospitals NHS Trust, Watford General Hospital, Vicarage Road, Watford, Hertfordshire, WD18 0HB UK

**Keywords:** Assessment, Radiographs, Young adult, Hip joint

## Abstract

Radiographic examination remains the mainstay of the initial assessment of the young adult hip; however, common parameters are required to assist in the formation of accurate diagnoses and appropriate management plans. This paper aims to summarise the most important aspects of the assessment of plain radiographs performed on the young adult hip joint.

## Introduction

An enhanced awareness of the presence of structural disorders of the hip, such as developmental dysplasia of the hip and femoroacetabular impingement (FAI), has fuelled an evolution in the assessment of patients with hip pain and enhanced our ability to diagnose patients, even in cases where there are mild structural abnormalities. Radiographic examination remains the mainstay of the initial assessment; however, common parameters are required to assist in the formation of accurate diagnoses and appropriate management plans including appropriate further imaging. This paper begins by describing the parameters that potentially impact the quality of antero-posterior (AP) and lateral radiographs of the hip, and the variations in lateral radiographs that can be used. This article aims to summarise the most important aspects of the assessment of plain radiographs performed on the young adult hip joint. Acetabular and femoral parameters that are assessed on plain radiographs are then described.

## Materials and methods

A literature review into the assessment of hip pain in young adults was performed on Medline and Embase using the search terms ‘hip pain’, ‘young adult’ and ‘plain radiographs’. Articles were then assessed for any references relating to assessment of hip pathology using plain radiographs with a view to summarising the key findings.

## Antero-posterior view

A plain antero-posterior (AP) radiograph of the pelvis facilitates an assessment of each hip joint on an individual basis, as well as allowing for a comparison to be made to the contra-lateral hip joint. The AP pelvis X-ray can be performed in a supine position, with efforts made to control the pelvic tilt and rotation, enabling similar radiographs to be obtained in every patient. It can also be obtained in a weight-bearing manner, which may accentuate any arthritic changes, and give an indication of limb length discrepancy. When initially reviewing a plain radiograph of the pelvis, the radiograph should be assessed for adequacy. Images should allow for visualisation of the entire pelvis including the iliac crests, sacrum, sacroiliac joints, pubic and ischial rami, as well as the necks of the femora and the lesser and greater trochanters [1]. In addition, the following factors should be considered.

### Pelvis rotation

**Fig. 1:**
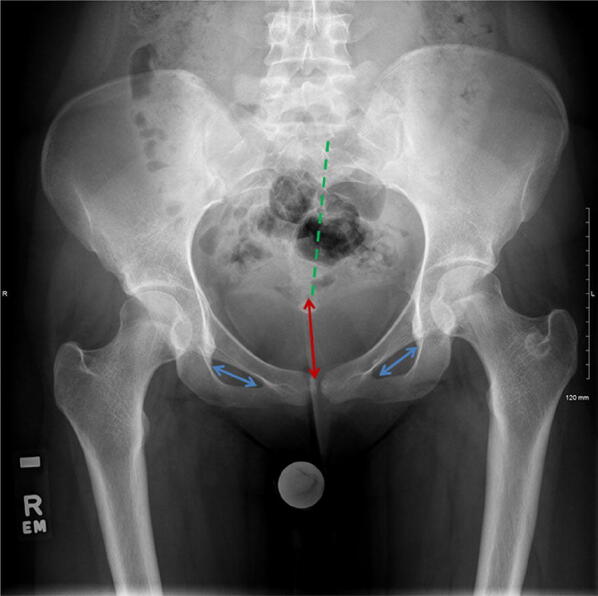
AP radiograph pelvis depicting pelvic rotation and tilt. The central sacral line (green dotted line) should be aligned centrally from the tip of the coccyx to the symphysis pubis. The obturator foramina (blue arrows), ischial spine and trochanters should be symmetrical. In this case, there is a slight pelvic rotation. The distance between sacro-coccygeal junction and the superior end of the symphysis (red arrow) is used to determine the pelvic tilt. A benign sclerotic ring is seen in the left trochanteric region

An acceptable plain radiograph of the pelvis should be obtained with pelvis in a neutral position. In the absence of pathology, the two obturator foramen, ischial spines, greater and lesser trochanters and femoral heads should be symmetrical. The central sacral line and the tip of the coccyx should also align with the symphysis pubis (Fig. 1).

### Pelvic tilt

A plain AP radiograph should have neutral tilt, which may require correction of lumbar lordosis. The distance between the sacro-coccygeal junction and the superior end of the symphysis should be between 2 and 3 cm in males and between 2 and 6 cm in females [2] (Fig. 1).

### Rotation of the lower limb

Approximately 15° of internal rotation of the hips, such that the greater and lesser trochanters are parallel to the floor, maximises the length of the femoral neck and enables a more accurate neck-shaft angle measurement [3]. Acquisition of the radiographic image with the patellae facing anteriorly can lead to over-estimation of the true neck-shaft angle [4].

### Film exposure

The exposure of the radiograph is related the concentration of photons absorbed by the film, or digital detector, at the time the radiograph was taken. Radiographic contrast is defined as the density difference between neighbouring areas on a plain radiograph [5, 6]. Appropriate film exposure and contrast should enable delineation of the acetabular walls and the fat pads around the hip joint (gluteal, obturator and iliopsoas).

## Lateral radiographs

A lateral radiograph of the hip can provide valuable information in the assessment of a young adult with hip pain. A variety of lateral views have been developed and are described.

### Cross-table lateral view

**Fig. 2:**
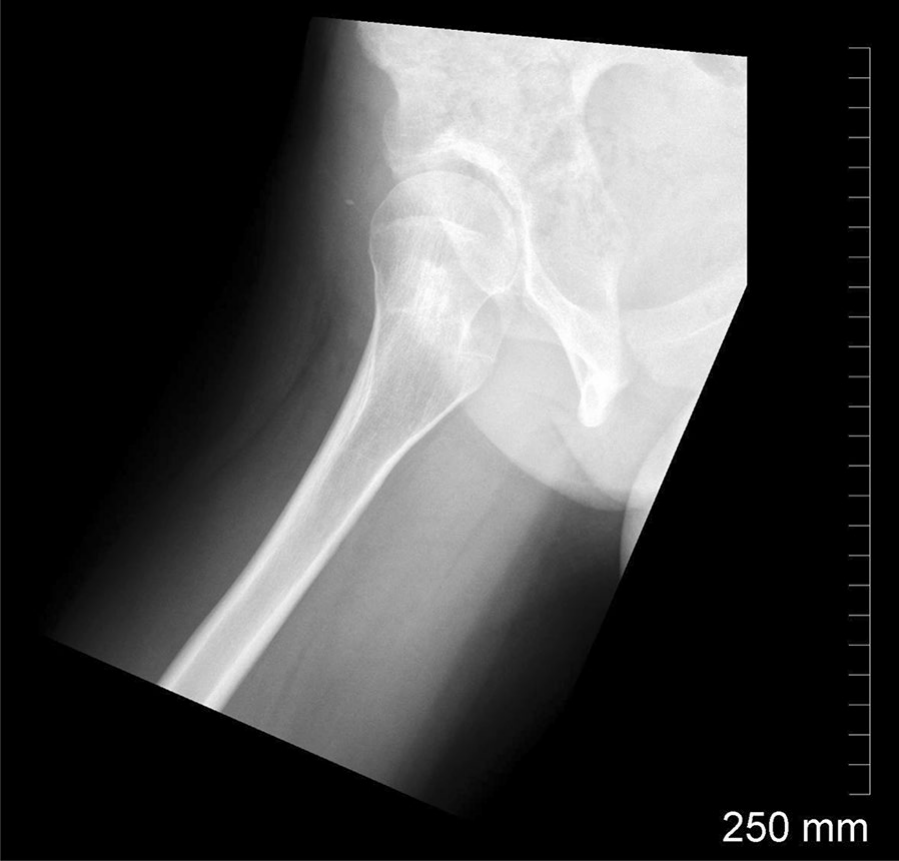
Cross-table lateral view

This view is performed with the patient supine on an X-ray table and the symptomatic limb in approximately 15° of internal rotation, and the image is taken at a 45° angle to the symptomatic limb (Fig. 2). The contra-lateral limb is flexed beyond 80° at the hip and knee joint. This view can demonstrate CAM deformities of the femoral neck and also gives clues about femoral version [7].

### False profile lateral view of the hip joint

**Fig. 3:**
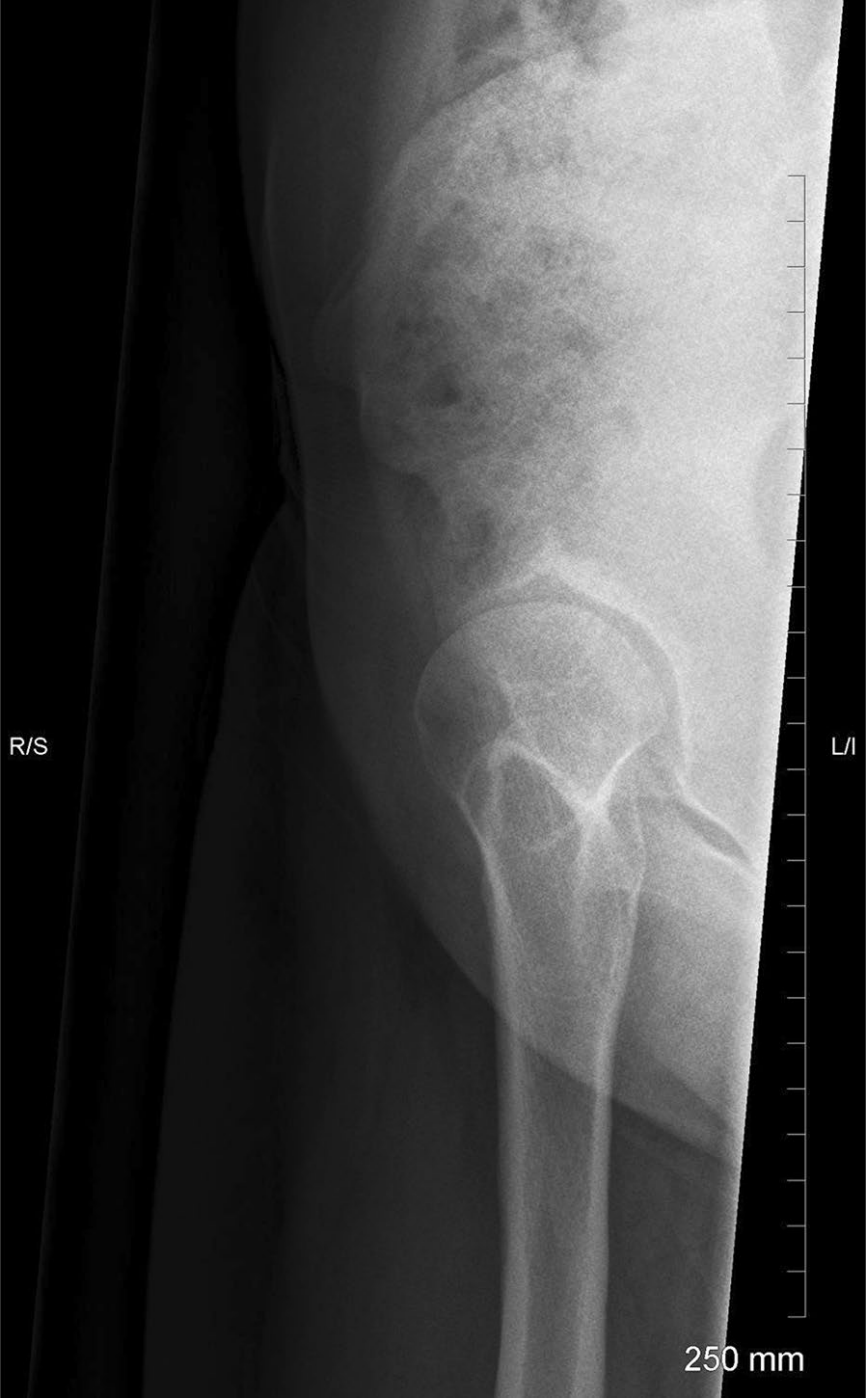
False profile lateral view

Taken with the patient in a standing position, it allows assessment of anterior acetabular coverage of the femoral head (Fig. 3) [8]. The anterior centre-edge angle can be measured, to investigate anterior subluxation during weight bearing. Only one hip can be studied at a time. This is especially useful when assessing a patient for peri-acetabular osteotomy (PAO).

### Frog lateral view of the hip joint

**Fig. 4:**
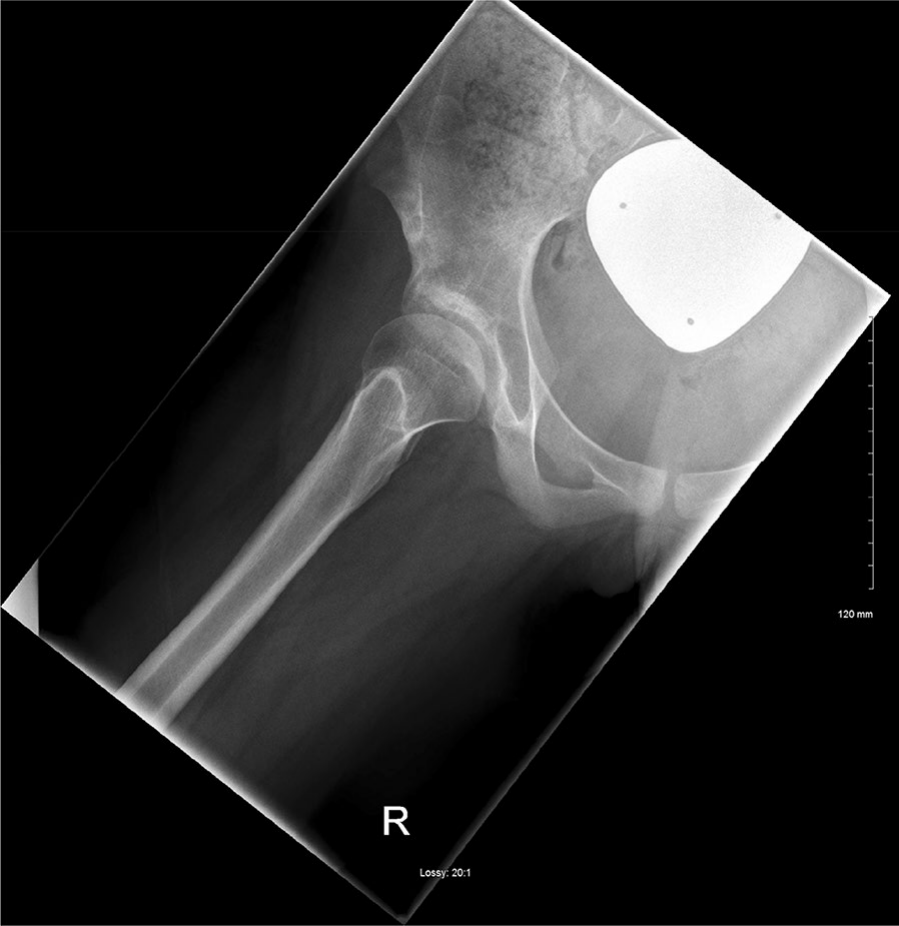
Frog lateral view

It is performed with the patient supine on the X-ray table with the hip externally rotated and abducted to 45°, and the knee flexed approximately 30°–40° (Fig. 4) [9]. With both hips visible, it is possible to compare the range of motion between the two. Pelvic tilt will not be controlled; therefore, acetabular morphology is difficult to analyse. This is more commonly used in the paediatric skeletally immature population.

### ° Dunn lateral view

**Fig. 5:**
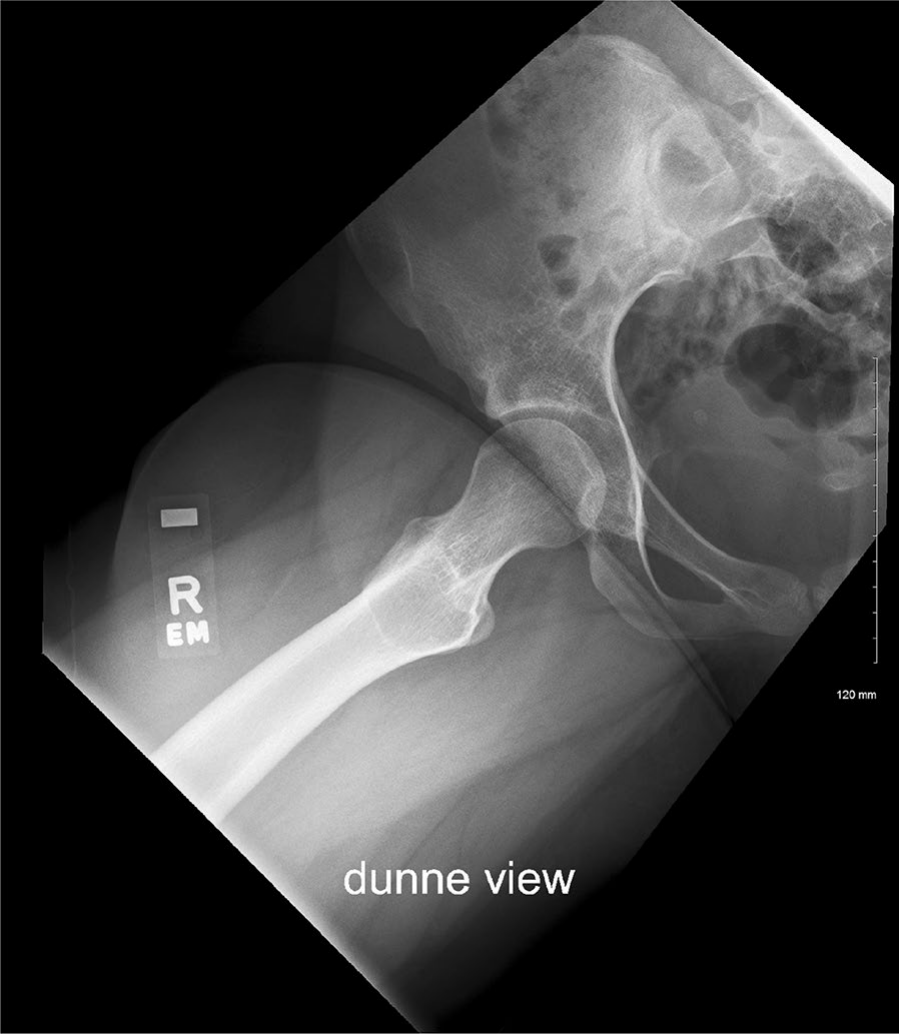
Dunn lateral view

The patient’s hips are flexed to 45°, in maximal abduction, with neutral rotation maintained providing a comparison image of both hips (Fig. 5) [10]. This view exhibits the anterograde superior head–neck junction well and allows femoral version to be estimated. It is also a helpful view for detections of signs of impingement.

### Modified Billing’s lateral view

A standard AP view of the hip is performed, but with 90° of flexion, 65° of abduction and neutral rotation of the hip. The leg can be supported by a wedge when obtaining this view [11, 12].

## Acetabular parameters

### Teardrop

**Fig. 6:**
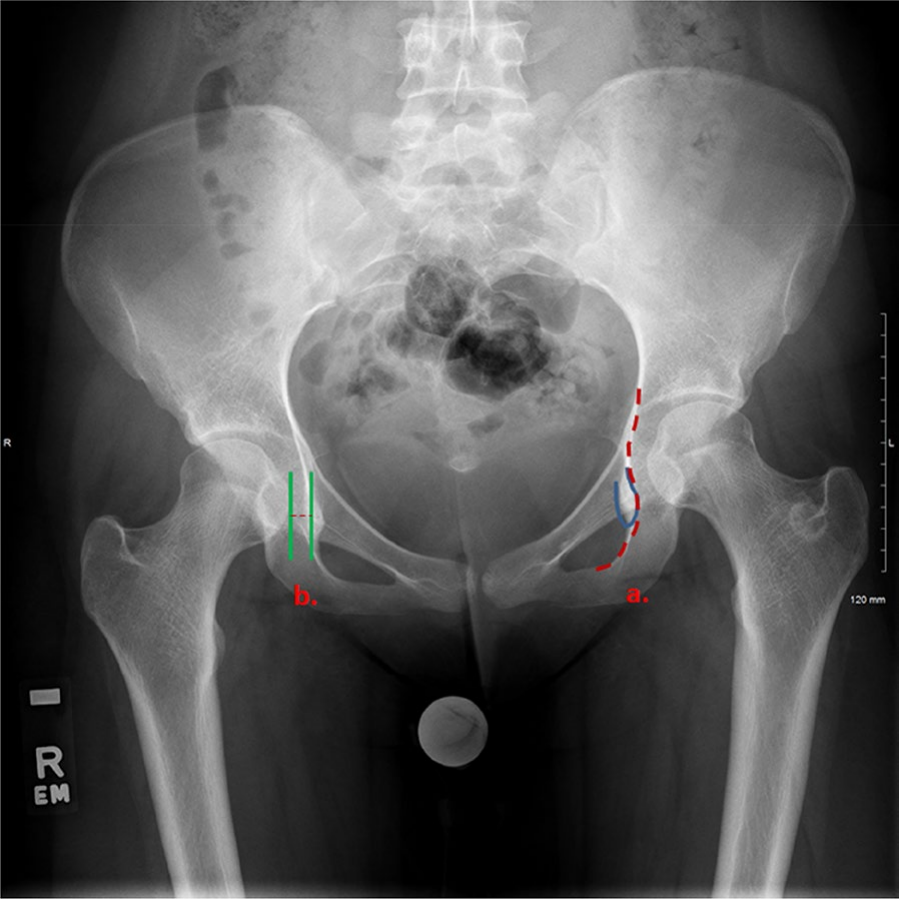
**a** Teardrop sign. The U-shaped (blue line) teardrop consists of ilioischial line (red dotted arrow) and floor of the acetabulum. **b** Teardrop distance

This radiographic teardrop represents the innominate bone at the inferior end of the acetabulum [13, 14]. The teardrop is normally U-shaped and made up of a medial border (continuous with the ilioischial line) and a lateral border (continuous with the floor of the acetabulum). A wider teardrop sign can typically represent acetabular dysplasia or joint effusion [15, 16], whereas a narrowing, crossover of the medial and lateral borders can indicate an acetabulum that is deeper than normal, resulting in coxa profunda (Fig. 6).

### Sourcil

**Fig. 7:**
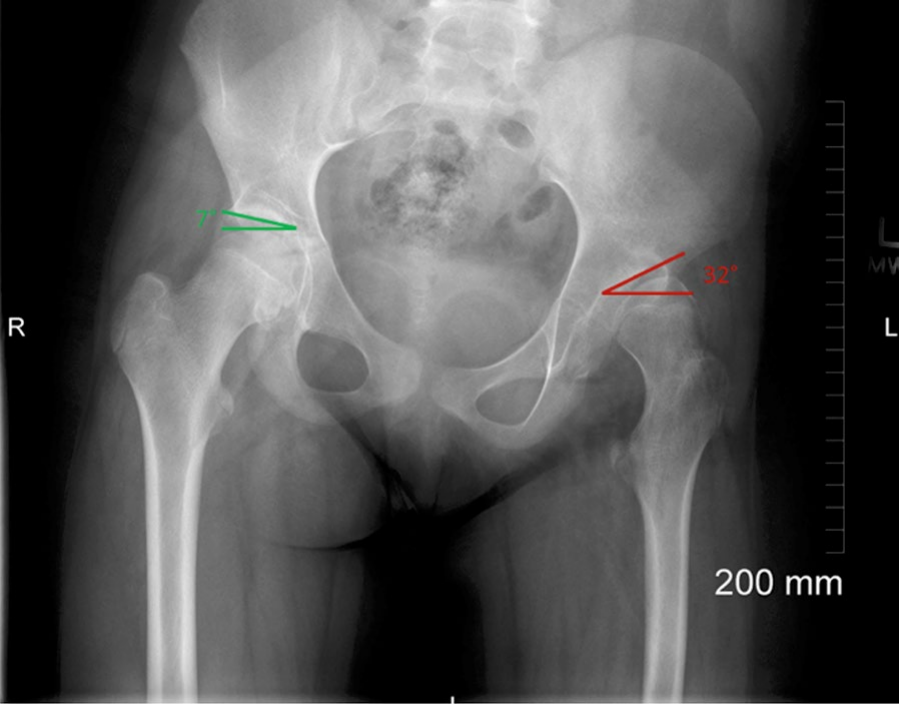
Sourcil (Tönnis) angle. The right hip (green angle) represents normal Sourcil angle, whereas the left hip (red angle) represents increased Sourcil angle. Lateral translation of the femoral head is clearly visible in keeping with hip dysplasia

This delineates the weight-bearing portion of the acetabular dome [17]. In a normal femoroacetabular joint, the Sourcil covers approximately 80% of the femoral head (difficult to assess in the presence of a CAM deformity). Acetabular inclination can be assessed on an AP Pelvic view using the Sourcil or Tönnis angle [18] (Fig. 7). These measurements can indicate dysplasia of the acetabular dome, whether a patient is at a higher risk of instability, or demonstrates risk factors for PINCER impingement.

The Tönnis angle is determined by drawing three lines:

1. A horizontal line connecting the base of the acetabular teardrops
2. A horizontal line parallel to line 1, running through the most inferior point of the sclerotic acetabular Sourcil
3. A line extending from most inferior to the lateral margin of the acetabular Sourcil.

The Sourcil, or Tönnis angle, is formed by the intersection of lines 2 and 3. This angle determines the joint reaction force that is transmitted along the primary compression trabecula of the femur perpendicular to the slope of the Sourcil.

An increased Tönnis angle (> 10°) increases the risk of lateral translation of the femoral head in relation to the acetabulum, which is initially contained by the labrum and joint capsule. Lateral subluxation of the head results when the labrum eventually fails. A downsloping Sourcil leads to medial translation of the femoral head and medial osteoarthritis or PINCER impingement.

### Acetabular version

**Fig. 8:**
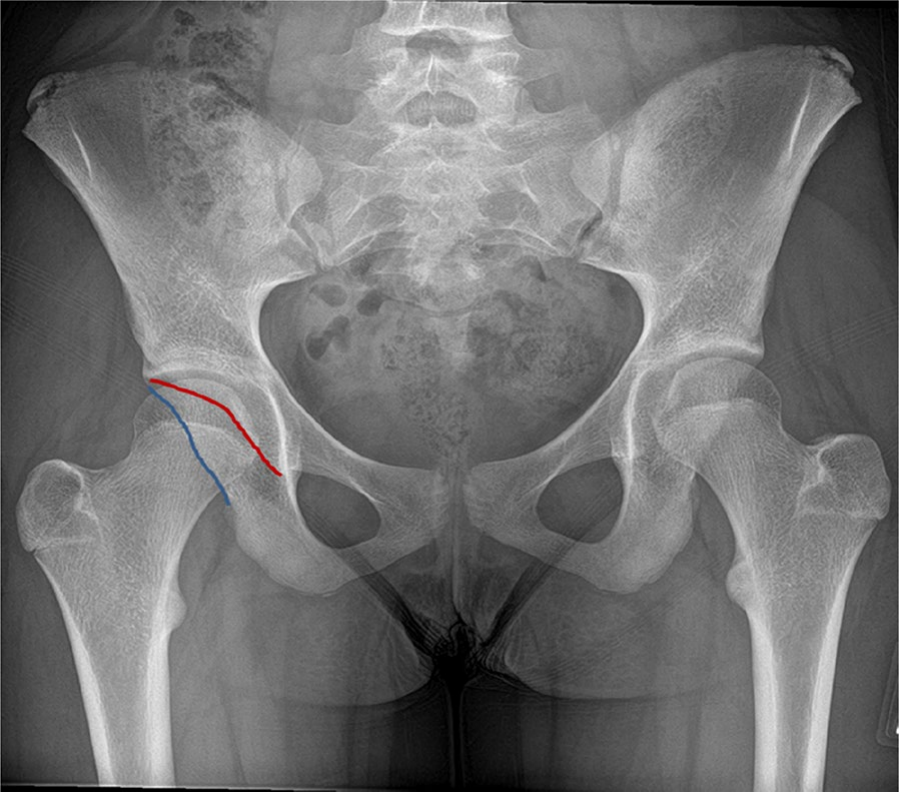
Anterior and posterior acetabular walls do not crossover and are only in contact at the lateral edge of the Sourcil suggestive of an anteverted acetabulum

Normal acetabulum version is approximately 20° of anteversion, and although difficult to accurately delineate on X-ray, it is best assessed on an AP view of the pelvis [19]. All acetabula can be labelled as retroverted or anteverted on the basis of the presence or absence of a crossover sign. The posterior wall of the acetabulum is relatively vertical and extends to the ischium, with the anterior wall being more horizontal and extending down towards the pubis. In an anteverted acetabulum, the anterior and posterior walls are in contact at the lateral edge of the Sourcil, but do not cross over more inferiorly (Fig. 8).

**Fig. 9:**
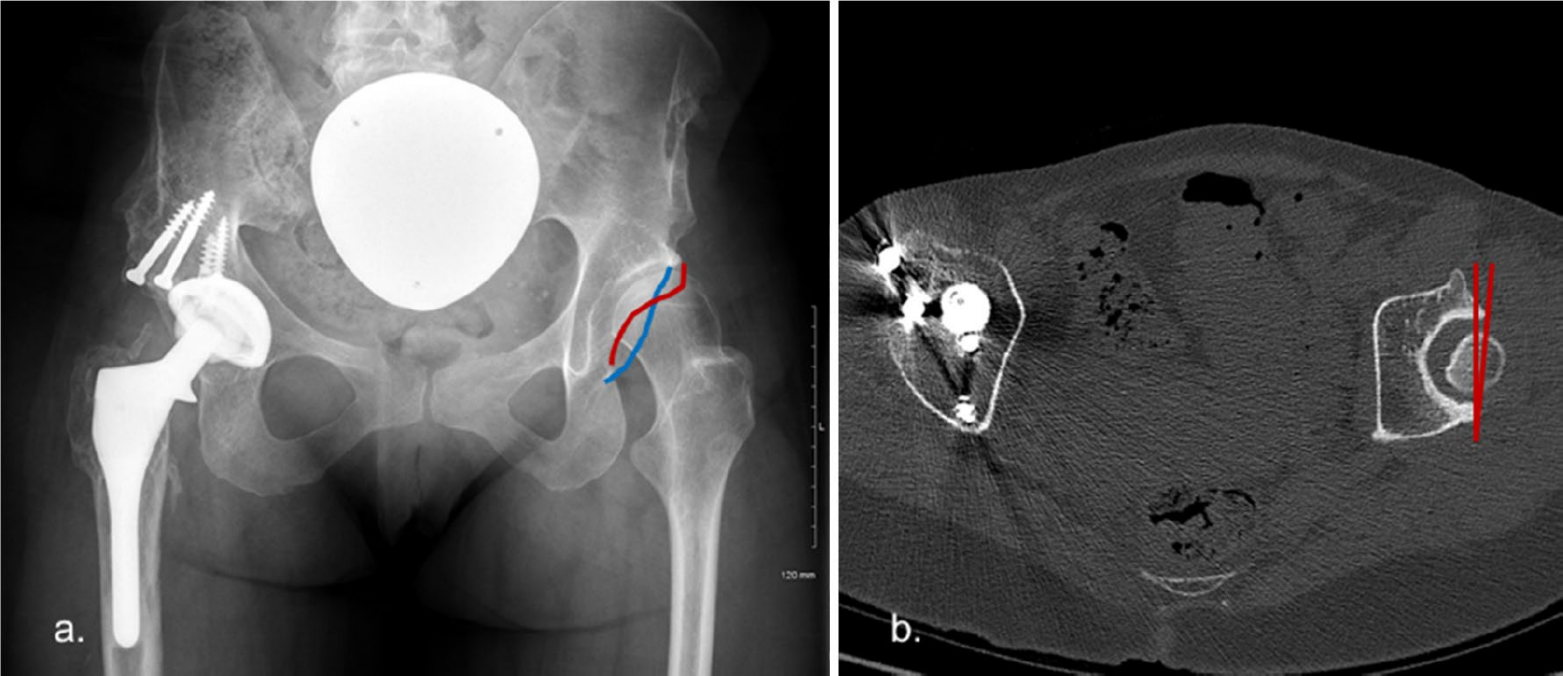
**a** AP radiograph pelvis. There is a crossover of the anterior and posterior walls of the left acetabulum, which represents acetabular retroversion. **b** CT axial image of the same patient which demonstrates left acetabular retroversion

The acetabulum is considered to be retroverted if the line representing the anterior aspect of the acetabular rim does cross the line representing the posterior aspect of the acetabular rim. This can be a difficult determination to make, requiring careful assessment of the film quality, needless to say CT scan is the more accurate method to demonstrate acetabular version (Fig. 9).

The line of the anterior wall of the acetabulum typically covers the femoral head to a lesser extent than the line of the posterior acetabular wall, which routinely passes through the centre of the femoral head. Passage of the posterior acetabular wall medial to the femoral head indicates that posterior coverage is deficient, whereas passage of the posterior acetabular wall lateral to the femoral head would indicate excessive posterior coverage [20].

### Lateral centre-edge (LCE) angle of Wiberg

LCE is another measurement of acetabular coverage of the femoral head. It is measured on the AP radiograph by forming an angle between a line through the centre of the femoral head, placed perpendicular to the pelvic tear drop line, and a second line placed along the lateral margin of the acetabulum. An angle between 25° and 40° is regarded as normal with 20°–25° being borderline. Ogata’s angle is a variant of the LCE angle where a line is drawn along the lateral edge of the sclerotic rim seen at the acetabular roof (Sourcil) instead of the lateral most edge of the acetabulum. Ogata et al. [20] have concluded that these are not the same in patients with developmental dysplasia of the hip (DDH) and concurrent acetabular retroversion. Placing a line along the lateral most edge of the acetabulum would likely lead to over-estimation of LCE angle in this patient group [21].

### Anterior centre-edge (ACE) angle

It is used to evaluate the anterior coverage of the acetabulum on false profile view as previously mentioned. A line is placed at the centre of the femoral head, which is parallel to the femoral neck on false profile view. A second line is placed along the anterior acetabular rim forming an angle. A normal angle would be between 20° and 40° with lesser and greater measurements representing anterior acetabular under and over coverage, respectively [22].

### Acetabular quotient

This ratio is dependent on the acetabular depth and acetabular width. The width of the acetabulum is measured between the inferior margin of the teardrop and the lateral margin of the acetabulum. A perpendicular line is placed from the midpoint of the acetabular width measurement to the centre of the acetabular dome. The measurement is performed on AP radiograph and calculated via acetabular depth/acetabular width × 1000. A value of less than 250 would be regarded as representing hip dysplasia [22].

Other radiographic signs to be aware of include the presence of subchondral cysts, which develop from rim loading and after advanced damage from CAM impingement. Osteophyte formation develops from the ossified labrum around the rim of the acetabulum in pincer impingement.

## Femoral parameters

The femoral head has a concave and largely symmetrical outline. Flattening of the femoral head or overgrowth of the epiphysis on to the femoral neck can produce a misshapen CAM-type impingement and result in incongruity between the femoral head and acetabulum.

### Sagging rope sign

On a AP view of the pelvis, the sagging rope sign identifies abnormal extension of the femoral head on to the femoral neck [23]. The extent of the deformity can be assessed on a cross-table lateral, a frog lateral or a 45° Dunn view, with Mose circles [24] utilised to objectively assess the sphericity of the femoral head. Clohisy et al. [3] state that if the femoral epiphysis extends beyond the margin of a reference circle by more than 2 mm, the femoral head is considered aspherical.

**Fig. 10:**
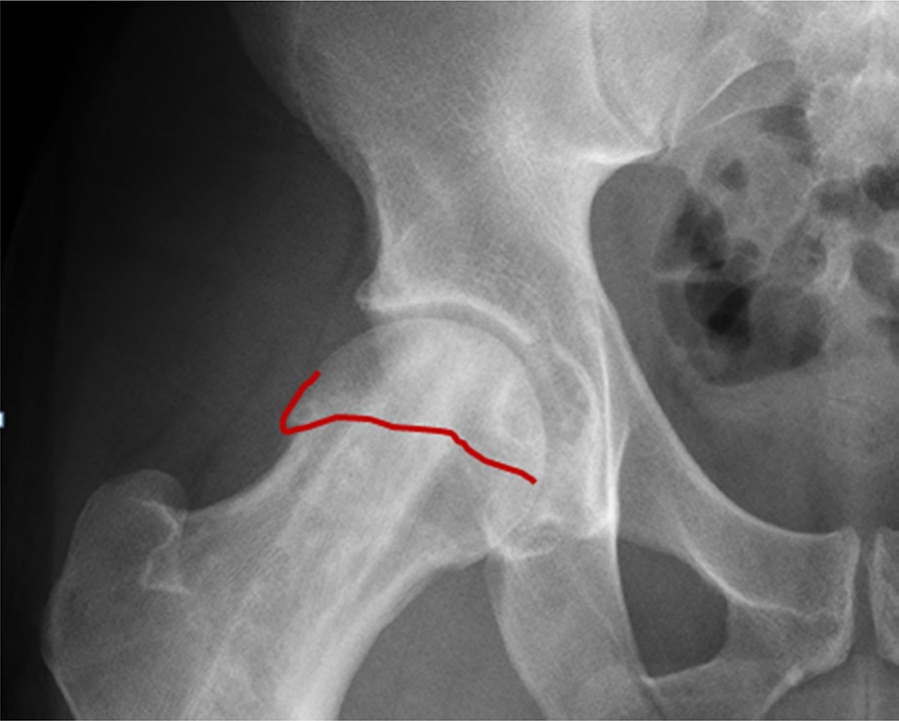
Red line represents a physeal scar which is seen to extend along the lateral margin of the femoral head–neck creating a CAM-type deformity

Extension of the physeal scar to the superior aspect of the femoral neck on an AP pelvis is typical of a CAM deformity of the femoral head (Fig. 10).

### Fovea centralis

**Fig. 11:**
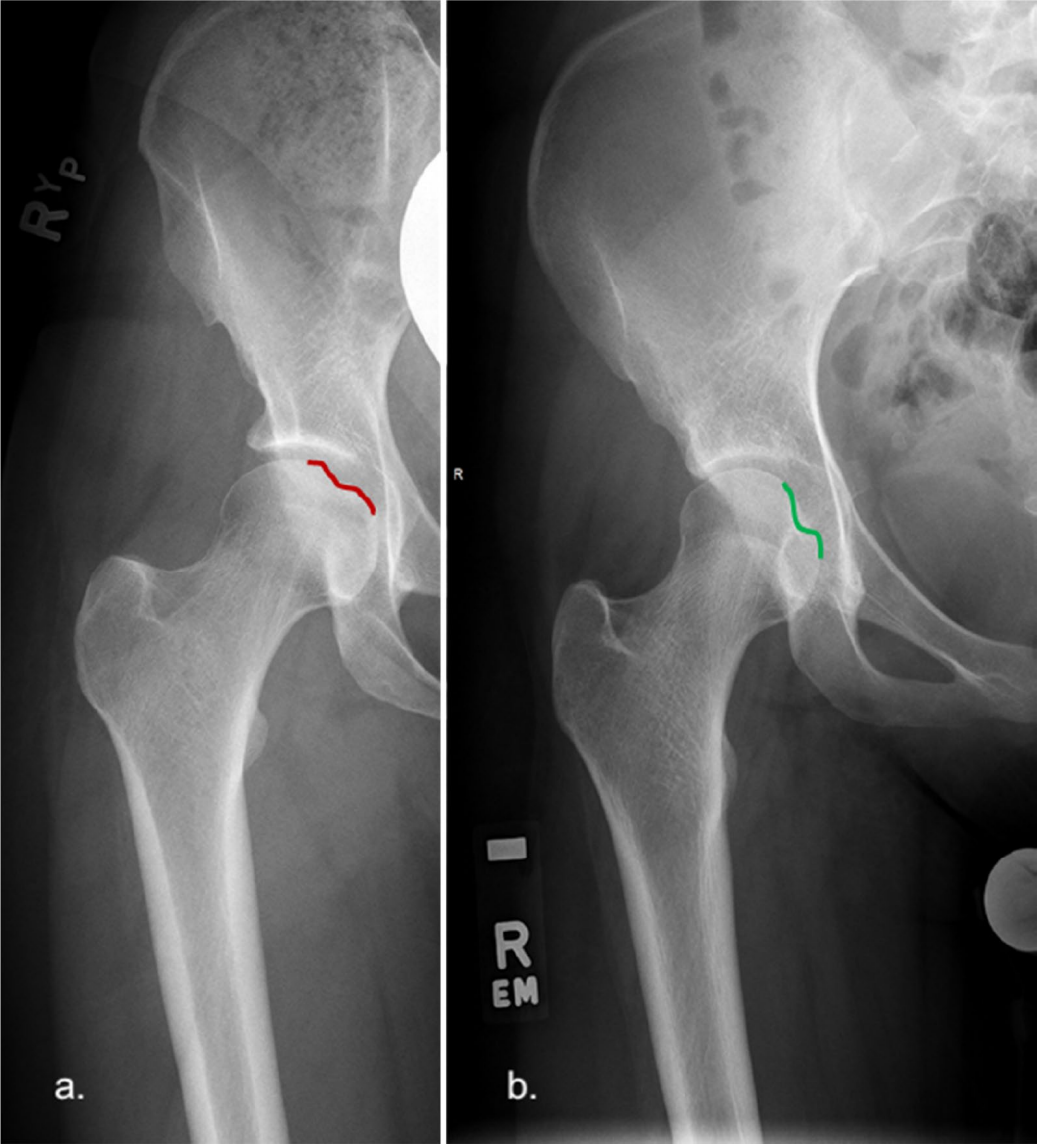
**a **Fovea on the right (red depression) is in contact with the Sourcil and represents Fovea alta. **b** Fovea on the left (green depression) is located medially and inferiorly and is therefore normally situated

The fovea is another sign that is visible on an AP pelvis radiograph. This represents the attachment of the ligamentum teres and represents somewhat of a watershed point. The articular cartilage inferior to the fovea is thinner in comparison with the cartilage superior to the fovea. The fovea typically manifests on a normal plain radiograph as a depression that is located medially and inferiorly, such that it does not come into contact with the Sourcil. A fovea that is superiorly placed (coming into contact with the Sourcil) is abnormal and is called a Fovea alta (Fig. 11) [25].

The femoral neck provides adequate clearance around the femoral head to allow a normal range of movement, but also provides a lever arm that allows the hip abductors to function normally. A number of variables can be identified in relation to the femoral neck on plain radiographs [26].

### Neck-shaft angle

**Fig. 12:**
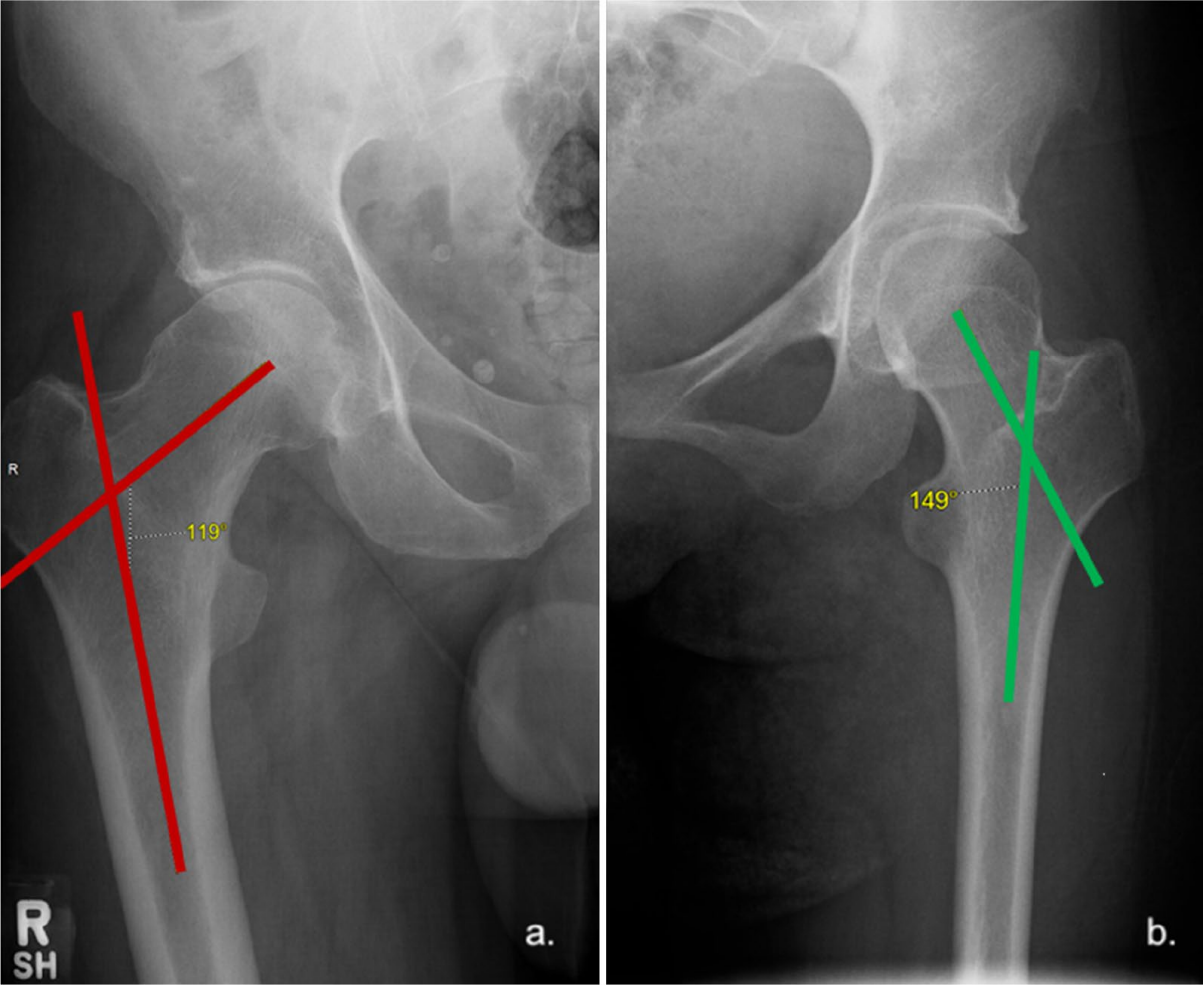
Neck-shaft angle. **a** Right hip (red angle) measures 119° and therefore is regarded as coxa vara. **b** Left hip (green angle) measures 149° and therefore represents coxa valga

The angle between the femoral shaft axis and the femoral neck axis is term the neck-shaft angle and is usually 125°–130° [27]. This angle can be calculated on an AP radiograph of the pelvis with the hips internally rotated approximately 15° (or until the femoral neck is horizontal to the floor). External rotation or excessive internal rotation of the femur will increase the apparent neck-shaft angle. Coxa valga is a deformity of the hip where the angle formed between the femoral neck and femoral shaft (neck-shaft angle) is increased, usually above 135°. Coxa valga can lead to increased joint reaction forces within the hip. Coxa vara is a deformity where the neck-shaft angle is less than 120° (Fig. 12). Coxa breva describes deformity where the femoral neck is excessively short. Coxa vara and coxa breva have the effect of reducing the resting length of the abductor muscles, which can manifest by limiting abductor function, resulting in a Trendelenburg gait [28].

### Femoral version

**Fig. 13:**
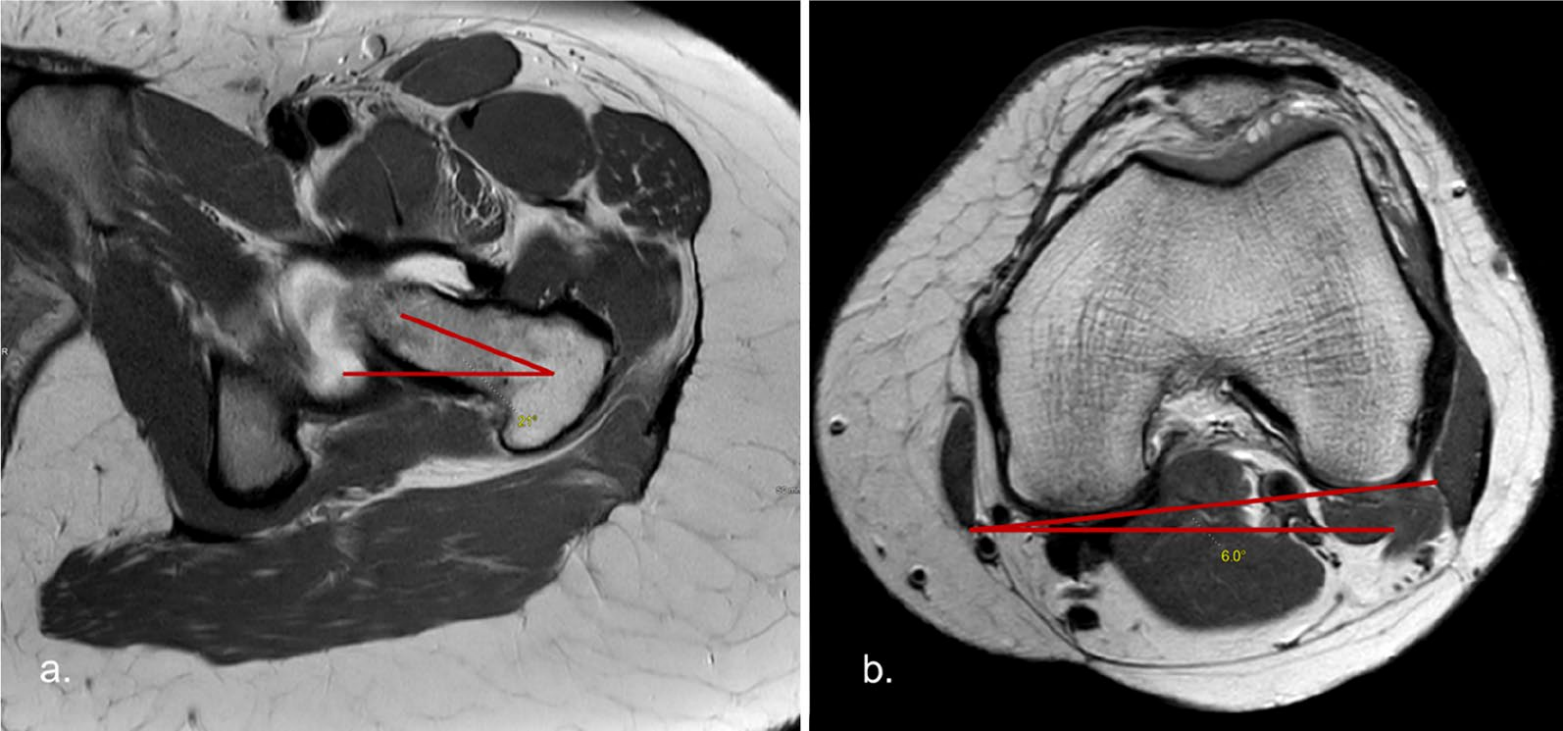
MR axial proton density (PD) sequences. **a** Level of the left femoral neck demonstrating femoral neck-horizontal angle. **b** Trans-condylar axis of the left knee demonstrating trans-condylar horizontal angle

It is described as the angular difference between the femoral neck-horizontal angle and the trans-condylar horizontal angle of the knee. When the femoral condyle is internally rotated, the trans-condylar horizontal angle is added to the neck-horizontal angle to calculate the degree of anteversion (Fig. 13). Normal version is approximately 5°–20° of anteversion [29].

### Femoral offset

It is measured as the perpendicular distance from the axis of the shaft of the femur to the centre of rotation of the femoral head and reflects the displacement of the femur from the pelvis. Femoral offset influences various factors associated with hip function including abductor muscle function and impingement [30].

A narrow femoral neck around a larger femoral head is desirable to maintain a good range of movement. Movement within the hip joint will be permitted until the acetabular labrum interacts with the femoral neck at its maximal concavity. The anterior femoral neck is most commonly the area of impingement, and this occurs when the abnormal head–neck offset [31] is more superior on the anterior aspect of the neck.

The head–neck offset ratio can be calculated using a lateral radiograph of the hip joint. Three lines need to be drawn:

1. A line through the long axis of the femoral neck
2. A line parallel to line 1 through the most anterior aspect of the femoral neck
3. A line parallel to line 2 through the most anterior aspect of the femoral head.

The distance between lines 2 and 3 is measured. This value is then divided by the diameter of the femoral head [32], with a ratio < 0.17 indicating the presence of a cam deformity.

### Alpha angle

**Fig. 14:**
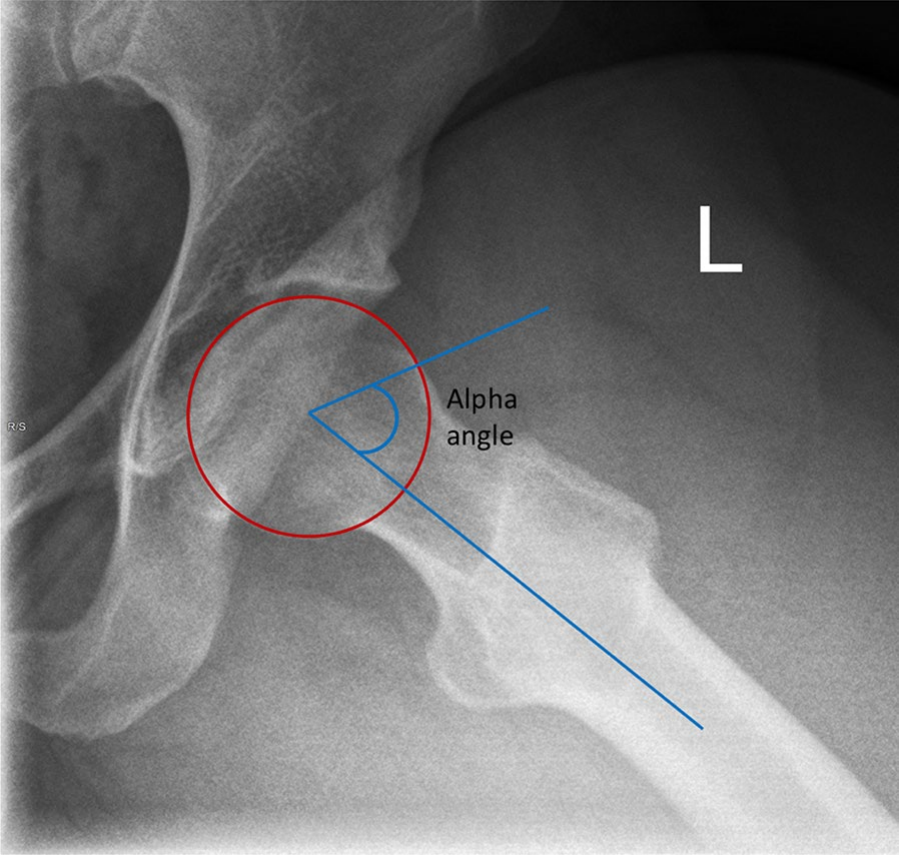
Alpha angle on lateral plain radiograph of left hip

The alpha angle was classically described for use with cross-sectional imaging (ultrasound, magnetic resonance imaging or computed tomography scans); however, a value can be extrapolated using a lateral radiograph of the hip joint (Fig. 14) [33].

Two lines are required to measure the alpha angle:

1. A line from the centre of the femoral neck to the centre of the femoral head.
2. A line from the centre of the femoral head to the point where the prominence starts (i.e. the point on the anterolateral aspect of the head–neck junction where the radius of the femoral head first becomes greater than the radius of the femoral head found more centrally in the acetabulum). Values of > 57° are suggestive of a head–neck offset deformity.

### Congruity of the hip

The articular surfaces of the femoral head and acetabulum are usually parallel to one another. Congruity of the hip can be assessed using all of the radiographic views described previously. The hip joint can be classified as congruous or incongruous based on the subjective assessment of the degree of conformity between the femoral head and the acetabulum.

### Hip centre

This can be assessed on an AP radiograph of the pelvis and can be classified as lateralised or not lateralised. This assessment is based on the position of the medial aspect of the femoral head in relation to the ilioischial line. A value greater than 10 mm denotes that the hip centre has been lateralised.

### Shenton’s line

**Fig. 15:**
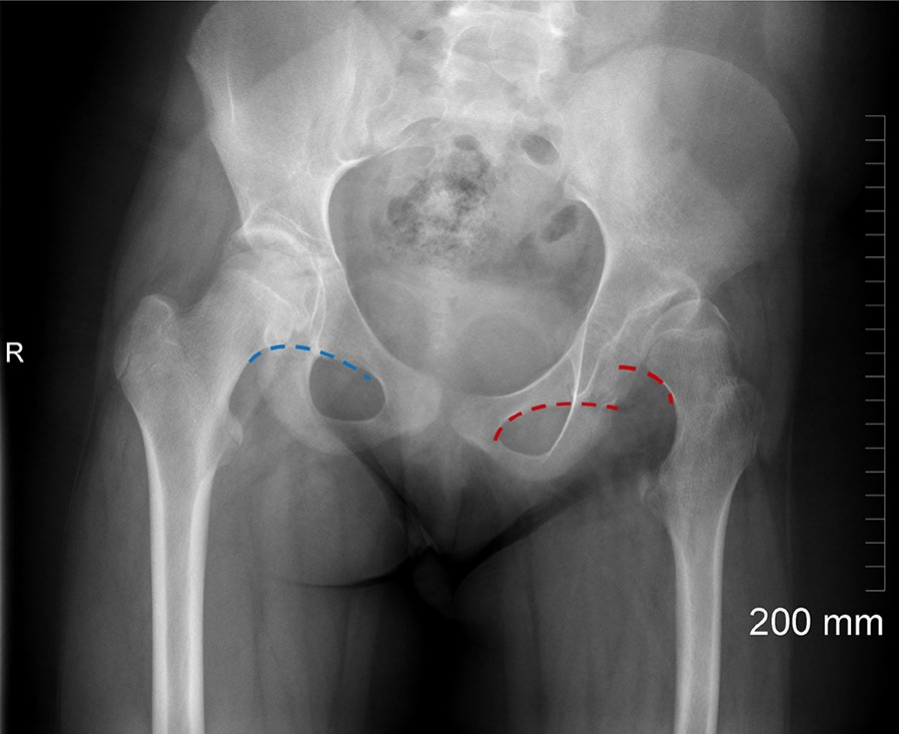
Right hip (blue dotted line) demonstrates preserved Shenton’s line. The left hip (red dotted line) represents ‘breaking’ of Shenton’s line resulting from superior and lateral subluxation of the dysplastic hip joint

This is an imaginary line drawn along the infero-medial aspect of the femoral neck and along the inferior border of the ipsilateral superior pubic ramus [34]. Superior and lateral subluxation of the femoral head will manifest on an AP radiograph of the pelvis by ‘breaking’ Shenton’s line (Fig. 15). Excessive external rotation of a normal hip can also break Shenton’s line without subluxation of the femoral head.

## Conclusion

An approach to the assessment of plain radiographs in young adult patients that present with hip pain has been outlined. Such an approach is required to adequately and reliably recognise the features that can be present in cases at the milder end of the spectrum in relation to structural abnormalities and will assist the diagnostic and decision-making process in this patient group. Further imaging modalities are usually essential in further assessing the young adult hip and can be utilised to confirm a suspected diagnosis and contribute to the appropriate management plan.
